# One Hundred Consecutive Cases of Skin Disease

**Published:** 1891-08

**Authors:** M. B. Hutchins

**Affiliations:** Lecturer on Diseases of the Skin, Atlanta Medical College, Atlanta, Ga.


### ONE HUNDRED CONSECUTIVE CASES OF SKIN DISEASE.

### VII.

By M. B. HUTCHINS, M. D.,
Lecturer on Diseases of the Skin, Atlanta Medical College, Atlanta, Ga.

### TRICHOPHYTOSIS. (RINGWORM.)

Of the various forms of trichophytosis, or ringworm, of the skin there were six cases. Three of these belonged to the class "eczema marginatum," as formerly called, or tinea cruris. Began in scroto-femoral region spreading with "circular" (border concave towards origin, convex towards healthy skin) to thighs, along perineum to buttocks, or slightly over scrotum and pubic region. Left slightly scaly and brownish track behind. The border, usually well defined, raised, about one-eighth of an inch wide, papulo-vesicular or "vesiculo-squamous"—marked by presence of closely aggregated, more or less distinct, little crusts. Occasionally a few typical lesions of ringworm as seen on the general skin (as the face or neck) external to the border of the principal diseased surface. Patients complained of much itching at night. These cases were adults, this form of ringworm being the more common in grown people.

The other three were cases of ordinary ringworm of the skin. I believe the first of these uncertain as the case was not kept up with.

The second case (of lady, age twenty-nine) was very peculiar, but the diagnosis was sufficiently easy. On flexure of left wrist was a circular non-elevated "spot" the size of a dime, and touching this another about the diameter of a pea, borders broken at point of contact. Center "clear"—only slightly brownish; border well defined, and about one-tenth of an inch in width, of purplish color and seemingly a little depressed, as if deep in the skin. Irritation produced itching. Condition present fourteen months, having begun as "a pimple" which gradually spread, clearing in the center. The peculiar appearance, and absence of scaling, I attributed to the applications made by the patient and the liberal *washing* the patch received. The patch had almost disappeared when the patient left the city about ten days after beginning treatment.

This case illustrates the puzzling appearances one sees in skin disease modified by previous treatment, and it is always well to inquire what has been previously done for the case, as the local treatment may have so modified the condition that it is unrecognizable through descriptions in the books.

Third case was simple and typical. Patient, a little girl of seven. Forehead, face, axillæ and left wrist affected. Began as small scaly "spots" soon forming the typical rings, with which, I think, every physician is familiar. The little girl probably "caught it" at school, and a little brother and sister very soon also became affected.

In the treatment of ringworm I have found the prescription below very effective, convenient and satisfactory:

℞. Acid pyrogall., gr. xv.
Collodii., ℥i.

M. Sig.—Paint on and let dry, p. r. n.

An explanation of its action might appear purely theoretical, and, as these papers are intended to be only practical, I shall not enter into the theory of action of this prescription. The application frequently produces considerable irritation and may aggravate the itching, but under its use the typical lesions rapidly disappear. As the slightest remains of the fungus which produces the disease will cause it to relapse, no case should be left alone until there is absolutely no appearance of activity in the skin lesions, or until there is nothing left save a faint staining. If this rule is not followed you may hear months later that the disease kept relapsing "*and it must be in the blood.*"

IMPETIGO CONTAGIOSA.

It seems to me that Crocker, the English dermatologist, is right in his belief that the two usually described impetigos, and ecthyma should all come under the above heading. He attributes them to "pus infection," or, perhaps, to some not yet isolated micro-organism, producing pus. Thus, for example, I may mention the case of the young man reported to the Atlanta Society of Medicine last year (and report published in this Journal). The pustular lesions on the face were believed to have resulted from infection from a boil on the back of the neck. He recovered rapidly under cleanly and antiseptic treatment. General eharacteristics of these "impetiginous eruptions" were as follows:

Some cases showed pustules averaging the size of a pea, with red areola, sometimes a circle of raised epidermis, from serous exudation, between areola and base of pustule ; some older were crusted, the crust of yellowish brown color, superficial and easily removed. Other lesions were large, crusted, superficial, with red areola—ecthyma. As a rule single lesions formed rapidly, and in some cases were deep enough to produce faint scarring, but usually only slight pigmentation remained and this soon disappeared.

Favorite localization was on the face, hands, feet and buttocks, or it might be even more diffused. Opening the pustules immediately and the use of some one of the antiseptics, in "mild strength," as in the following, proved entirely efficacious:

℞. Ichthyol,
Zn. ox.,
Calamin. prep., aa ʒi.
Aquæ, ℥iv.
M. Sig. Apply frequently.

The number of these cases was five. Some had definite history of contagion, others doubtful. The localization, absence of grouping, and the symptoms given above sufficed for the diagnosis.

VITILIGO.

Of vitiligo, leucoderma or spotted (white) skin two cases were recorded. This is the disease producing the peculiar spotting of the skin in negroes who are sometimes exhibited in museums as "spotted people" or "negroes turning white." The two cases recorded were of white men, the first with a few small white spots beneath the jaw and quite large patches of non-pigmented skin on scrotum and penis. The other man had patches from reddish to clear white on face, neck and hands bordered by apparently deeper than normal pigmentation. This patient thought the trouble resulted from severe fright some fourteen years ago. Though only thirty-four he was as gray haired as a man of fifty; patient says it began to turn gray two weeks after the accident in which he was frightened and a limb broken. Prognosis in both cases was very discouraging. Attempt was made to "bring back" some of the pigment by the inflammatory action of cantharidal collodion, but the treatment was not persisted in. Perhaps the only thing to do in these cases is to devise something which will hide the white spots or make them nearly resemble the normal skin.

PSORIASIS.

There were two cases of this disease. First, vigorous young lady of seventeen. On legs below knees a number of roundish or circular, well-defined, circumscribed patches, with adherent scales, patches having parchment-like feel. On knees, elbows

(favorite location), and forearms, a few fingernail-sized spots covered with dry, "silvery" scales, which being scraped off exposed the bleeding papillæ — "punctate hemorrhage"— quite characteristic of psoriasis.

Under ℞. Ac. salicyl., gr. xv.
Collodii flex., ℥i.
M.

painted on, the small lesions were "about well" in a week. After three months, recovered under scrubbing patches with sapo viridis and alcohol, followed by application of

℞. Picis liq., ℥i.
Ether sulph.,
Sp. vin rect., aa ℥i.
M.

And taking "Fowler's solution" in dose gradually increased to tolerance. Relapse last August relieved in about ten days with same treatment.

Second case, that of a young doctor, who wrote a description of the case and sent some of the scales by mail. Diagnosis of psoriasis was made and treatment given, but I have not heard further from him. It is generally possible to remove the eruption of psoriasis, but an absolute cure is doubtful of attainment.

Epithelioma, ulcers, etc., next article.

1½ *Edgewood Avenue.*

---

ANTIPYRIN IN INFANTILE ENURESIS.—Dr. J. Bouisson (Theses de Lyon) states that the effect of antipyrin in the treatment of the enuresis nocturna of childhood is "simply marvelous." The remedy is exhibited in doses of ten grains, repeated to the third time (30 grains in all) at intervals of one hour, commencing four hours before bedtime. Of eight inveterate cases in which the disease had existed for several years, and upon which every other remedy and method of treatment had proved futile, every case was completely cured. Several months have elapsed since the treatment, and in no case has there been a relapse, nor have any symptoms of return been noted.—*Nat. Druggist.*

